# PfSET2 Is Involved in Genome Organization of *Var* Gene Family in Plasmodium falciparum

**DOI:** 10.1128/spectrum.03891-22

**Published:** 2023-01-05

**Authors:** Xuan Cao, Yuhao Wen, Ying Li, Xuying Ma, Qingqing Jing, Lubin Jiang, Gang Wei

**Affiliations:** a CAS Key Laboratory of Computational Biology, Shanghai Institute of Nutrition and Health, University of Chinese Academy of Sciences, Chinese Academy of Sciences, Shanghai, China; b CAS Key Laboratory of Molecular Virology and Immunology, Institute Pasteur of Shanghai, University of Chinese Academy of Sciences, Chinese Academy of Sciences, Shanghai, China; Hubei University of Medicine

**Keywords:** malaria, *Plasmodium falciparum*, PfSET2, 3D genome, epigenetic, transcriptional activity, transcription

## Abstract

The three-dimensional (3D) genome structure of human malaria parasite Plasmodium falciparum is highly organized and plays important roles in regulating coordinated expression patterns of specific genes such as virulence genes which are involved in antigenic variation and immune escape. However, the molecular mechanisms that control 3D genome of the parasite remain elusive. Here, by analyzing genome organization of P. falciparum, we identify high-interacting regions (HIRs) with strong chromatin interactions at telomeres and virulence genes loci. Specifically, HIRs are highly enriched with repressive histone marks (H3K36me3 and H3K9me3) and form the transcriptional repressive center. Deletion of *PfSET2*, which controls H3K36me3 level, results in marked reduction of both intrachromosomal and interchromosomal interactions for HIRs. Importantly, such chromatin reorganization coordinates with dynamic changes in epigenetic feature in HIRs and transcriptional activation of *var* genes. Additionally, different cluster of *var* genes based on the pattern of chromatin interactions show distinct transcriptional activation potential after deletion of *PfSET2.* Our results uncover a fundamental mechanism that the epigenetic factor PfSET2 controls the 3D organization of heterochromatin to regulate the transcription activities of *var* genes family in P. falciparum.

**IMPORTANCE** PfSET2 has been reported to play key role in silencing *var* genes in Plasmodium falciparum, while the underlying molecular mechanisms remain unclear. Here, we provide evidence that PfSET2 is essential to maintain 3D genome organization of heterochromatin region to keep *var* genes in transcription repressive state. These findings can contribute better understanding of the regulation of high-order chromatin structure in P. falciparum.

## INTRODUCTION

Malaria is a life-threatening disease caused by *Plasmodium* parasites, with 241 million cases and an estimated 627,000 deaths worldwide in 2020 (1). P. falciparum is the deadliest species of five *Plasmodium* species that cause malaria in humans. *Plasmodium* parasites expressed variant antigen encoding erythrocyte membrane protein 1 (PfEMP1) at the surface of host red blood cells to escape the human immune system (2). PfEMP1 is encoded by approximately 60 *var* genes and only one *var* gene is expressed during the 48h replication cycle in red blood cells (3, 4).

Emerging lines of evidence have shown that epigenetic mechanisms play important roles in regulating genes expression in P. falciparum (5–18). Repressive epigenetic mark H3K9me3 is highly enriched in the silent *var* genes and other virulence families, such as *rifin*, *stevor*, and *pfmc-2tm* (5–7, 10). Heterochromatin Protein 1 (PfHP1), specifically recruited by H3K9me3, regulates the formation of heterochromatin and maintains the silence of *var* genes (19–21). Depletion of PfHP1 leads to *var* genes activation and parasite growth arrest at the trophozoite stage (20). Another epigenetic regulator, PfSET2, which controls H3K36me3 level mainly at *var* gene loci, also has repressive function in silencing *var* genes (10). Loss of PfSET2 results in marked reduction of H3K36me3 enrichment at *var* genes loci and transcriptional activation of most *var* genes.

More recently, studies have revealed that P. falciparum genome is highly organized in three-dimensional (3D) structure, with centromeres and telomeres clustered on opposite sides of the nucleus (22–28). *Var* genes, located at subtelomeric and internal regions, show strong interactions with telomeres clustering. FISH experiments also confirm that *var* genes form a few clusters around the parasite nucleus (7, 23, 29). Previous studies have shown that heterochromatin marks H3K9me3 and H3K36me3, as well as PfSET2 and PfHP1, are localized at nuclear periphery regions, including centromeres and telomeres (10, 11, 19, 20). This higher order organization of heterochromatin structure is important for the silencing of most *var* genes and is also regulated by some epigenetic factors. For example, depletion of PfHP1 results in greatly reduced chromatin interactions between *var* genes and loss of *var* genes repression (23). Because PfSET2 is also required for the maintenance of the repressive heterochromatin environment of *var* genes loci, it is important to determine whether PfSET2 is involved in regulating 3D chromatin structure (10).

In this study, we generate Hi-C maps for wild-type and *PfSET2*-KO samples at ring stage in P. falciparum (10, 30, 31). Analysis of these data sets, together with H3K9me3 and H3K36me3 ChIP-seq and gene expression data, allow us to uncover how 3D genome structure is regulated with the epigenetic and transcriptional changes at *var* gene loci in the parasite. Deletion of *PfSET2* results in change of telomeres clustering and marked reduction of H3K36me3 enrichment and chromatin interactions at *var* gene loci. Organization of *var* genes promoter regions in different groups reveals the precise regulation in transcription by 3D organization. Overall, our study provides insight into how epigenetic factor PfSET2 regulates the chromatin organization associated with the repression of *var* genes in P. falciparum.

## RESULTS

### Identification and characterization of high-interacting regions.

**FIG 1:**
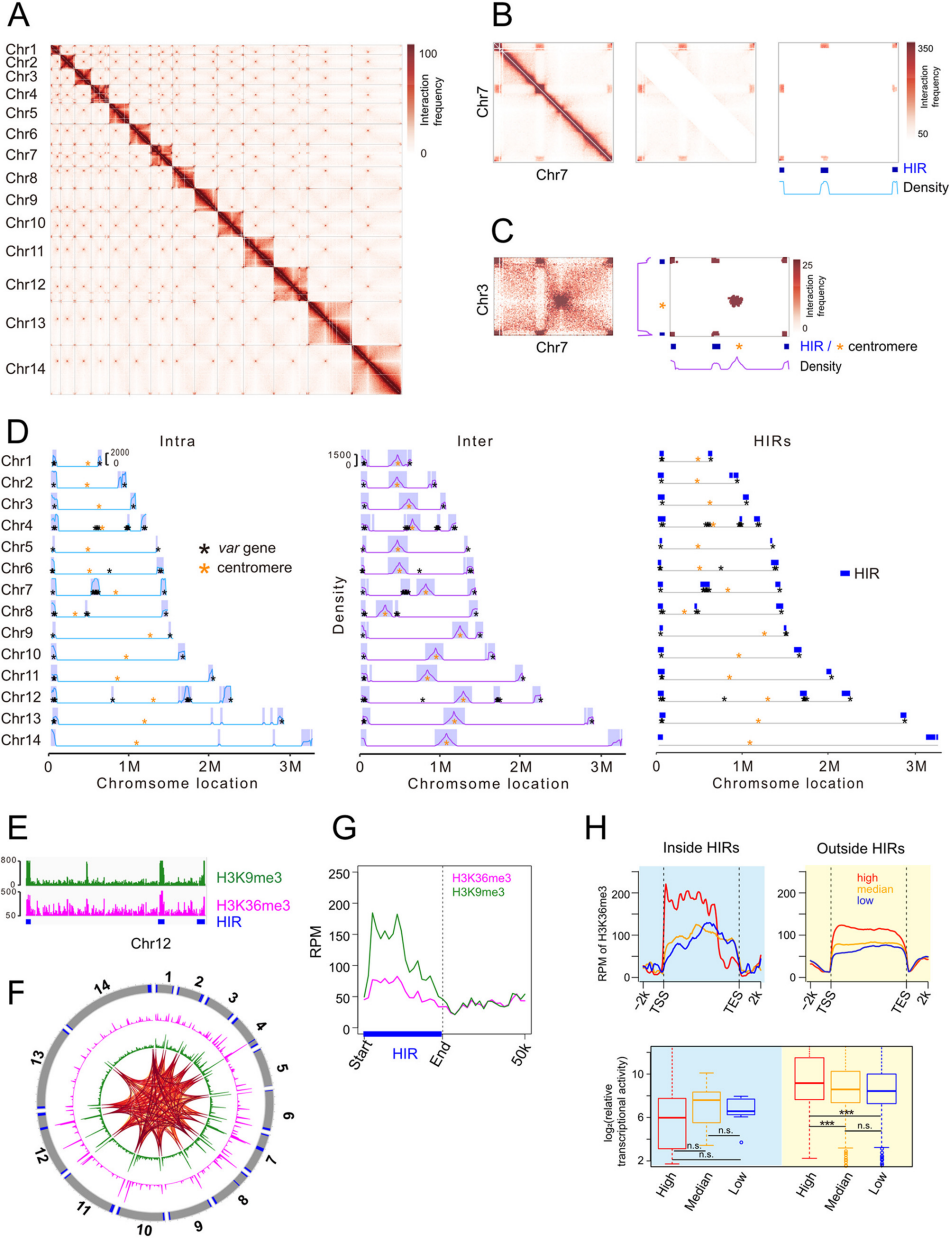
Identification and characterization of high-interacting regions (HIRs). (A) ICE-normalized whole-genome contact count matrix at 10 kb resolution. Individual chromosomes are delineated by lines. (B) HIRs calling for intrachromosomal of Chr7. Left: contact matrix of Chr7. Middle: contact matrix after removing the signal along the diagonal. Right: contact matrix for continuous interacting bins. HIRs are indicated with blue rectangles. Blue line represents density of contacts. (C) HIRs calling for interchromosomal contact of Chr3 and Chr7. Left: contact matrix of Chr3 and Chr7. Right: matrix for continuous interacted bin. HIRs are indicated with blue rectangles, centromeres with yellow *. Purple lines represent density of contacts. (D) Genome-wide locations of HIRs. Left and middle: shadows indicate high level intra- and inter- chromosomal interaction regions, blue lines and purple lines are contact frequency. Right: Genomic locations of HIRs. *Var* genes loci are indicated with black *, centromeres with yellow *. (E) Enrichment of H3K9me3 (green) and H3K36me3 (magenta) at Chr12. (F) Circos plots depict the strong H3K36me3 (magenta) and H3K9me3 (green) occupancy at HIRs loci. Orange lines represent HIR-to-HIR interactions. Chromosome numbers are given at the periphery of the circle. Blue rectangles at the circle represent HIRs. (G) Distribution of H3K36me3 and H3K9me3 signal at HIRs. (H) The H3K36me3 enrichment profile (top) and relative transcriptional activity (bottom) of genes inside (blue shadow) and outside (yellow shadow) HIRs in each group. Genes enriched with H3K36me3 were classified into 3 groups based on H3K36me3 signal at TSS~100bp regions. All box plots depict the first and third quartiles as the lower and upper bounds of the box, with a thicker band inside the box showing the median value and whiskers representing 1.5× the interquartile range.y regions.

We performed Hi-C experiments at ring stage with two biological replicates that each sequenced 46 to 70 million paired-end tags, generating 18 to 28 million unique valid tags (30, 31). As shown in Fig. S1, the replicates had high reproducibility and were combined for subsequent analysis (32). Previous works have shown that the P. falciparum genome is highly organized in 3D structure, with strong chromatin interactions in centromeres, telomeres, and virulence genes loci (22–24, 26–28). Consistent with these reports, examination of the genome-wide contact matrix from our Hi-C data revealed strong interactions between centromeric regions and between telomeric regions (Fig. 1A). When we examined the intrachromosomal interactions in chromosome 7, two telomeric regions and one internal region exhibited high density of interaction frequency (Fig. 1B). When we checked the interactions between chromosome 7 and chromosome 3, the three regions with high density of intrachromosomal interaction also showed strong interchromosomal interactions with two telomeric regions in chromosome 3, while the two centromeric regions of these two chromosomes were highly interacted with each other (Fig. 1C). To investigate how these high-interacting regions were organized, we identified all the genomic regions in genome-wide with strong interactions of both intrachromosomal and interchromosomal and called these regions high-interacting regions (HIRs) (Fig. 1D). In total, 43 regions showed high intrachromosomal interactions and 57 regions exhibited strong interchromosomal interactions, generating 34 HIRs in P. falciparum genome at ring stage (Fig. S2A and B; Table S1). These HIRs were colocalized with all of 28 telomeres and 18 internal regions, with an average size of 65 kb (Fig. S2C). Notably, centromeric regions showed only high density of interchromosomal interaction, and thus, were not included in HIRs.

It has been reported that most of *var* genes are organized into close space to keep their repressive state (22–24, 26–28). Interestingly, 58 out of 62 *var* genes were found within HIRs, suggesting HIRs were generally located in heterochromatin regions (Fig. 1D; Fig. S2E). In addition to *var* genes, most of members of *stevor* and *rifin* gene families were included in HIRs (Fig. S2E). These genes showed much lower expression level compared with genes outside HIR regions (Fig. S2F). To further characterize the chromatin state of HIRs, we next examined the enrichment of several histone modification marks, including H3K9me3, H3K36me3, H3K4me3, H3K36me2, and H4K20me3 around HIRs. Two repressive chromatin marks, H3K9me3 and H3K36me3, are highly enriched at HIRs with sharp depletion at the boundaries and their flanking regions (Fig. 1E and F). In contrast, active chromatin mark H3K4me3 level was relatively lower at HIRs compared with the flanking regions (Fig. S2D). H3K36me3 has been considered a hallmark of actively transcribed regions while several studies have reported that H3K36me3 was also markedly enriched at pericentromeric heterochromatin in mouse and fission yeast (33, 34). To examine the role of H3K36me3 in transcriptional regulation, we classified genes enriched with H3K36me3 at TSS~100bp regions into three groups by H3K36me3 level (Fig. 1H, top). The enrichment level of H3K36me3 was negatively associated with transcription activity inside HIRs, while positively associated outside HIRs (Fig. 1H, bottom). In addition, genes inside HIRs showed much lower level of transcriptional activity than genes outside HIRs, suggesting that H3K36me3 mainly play a repressive role in repressing genes transcription inside HIRs. Taken together, these results suggest that HIRs, occupied by repressive histone marks, could be involved in heterochromatin organization in regulating virulence genes silence.

### PfSET2 regulates chromatin interactions of HIRs.

**FIG 2:**
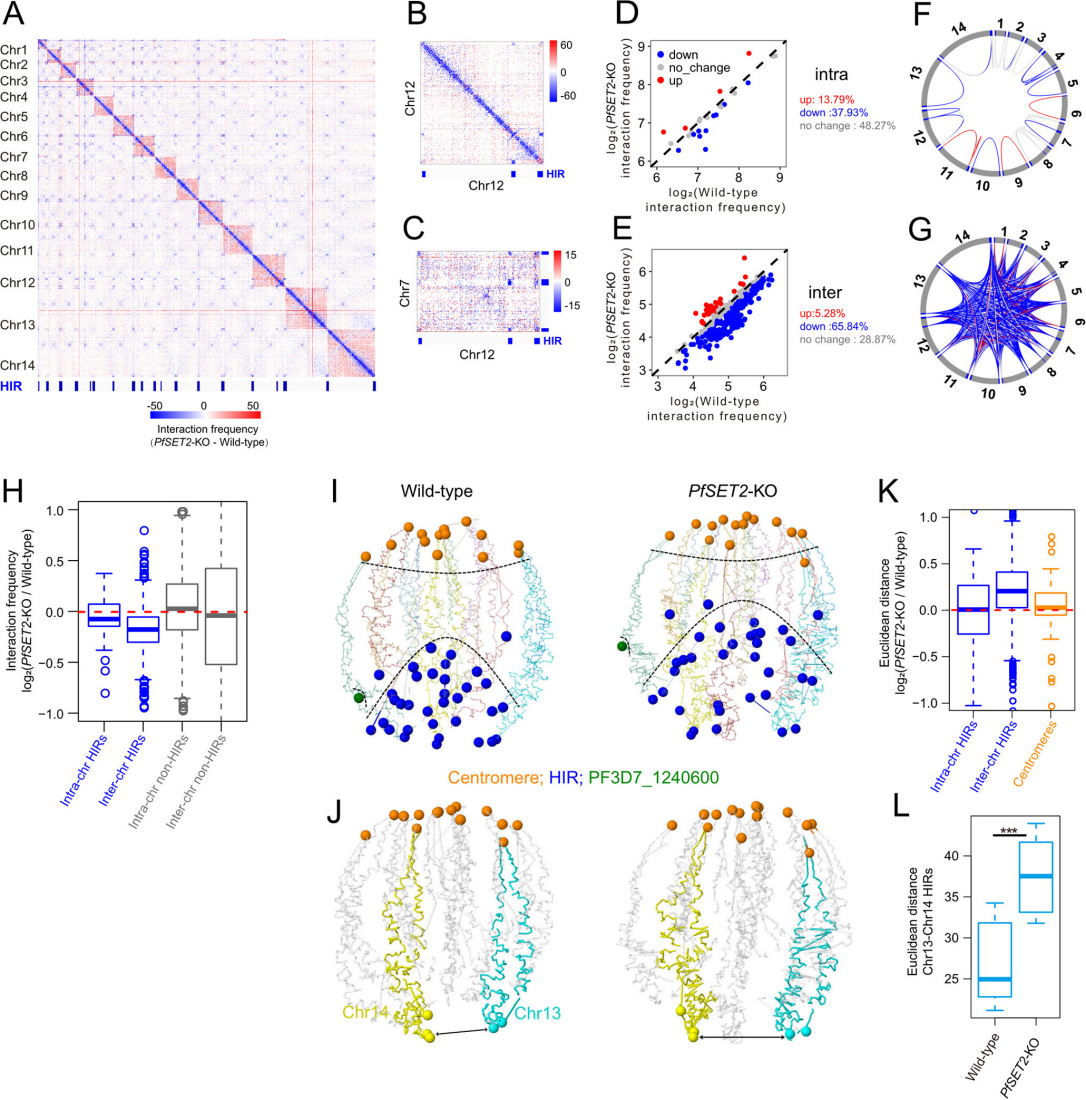
Deletion of *PfSET2* disrupts chromatin interactions between HIRs. (A) Genome-wide differential contact map at 10 kb resolution reveals reduced contacts between HIRs (blue rectangle) after *PfSET2* deletion, plotted as minus (contacts in *PfSET2*-KO - wild-type). (B) and (C) Differential contact map for intrachromosomal of chr12 and interchromosomal of chr7 and chr12. (D) and (E) Scatterplots of contact frequency made by HIR-to-HIR interactions located in intra (*n* = 29) and inter (*n* = 568) between wild-type (*x* axis) and *PfSET2*-KO (*y* axis) samples. HIRs with reduced interaction frequency in *PfSET2*-KO are represented in blue, increased in red and no changed in gray. Numbers of percentage are shown on the right of the scatterplot. (F) and (G) Circos plots depicting the contact frequency changes of intra and inter HIR-to-HIR interactions. Lines' colors are as in (D). (H) Box plots showing reduced contacts frequency for both intra and inter HIR-to-HIR interactions comparing *PfSET2*-KO with wild-type samples while median values of non-HIR-to-non-HIR are closed to zero. (I) 3D models for wild-type and *PfSET2*-KO samples. Chromosomes are shown as transparent ribbons with different colors. Centromeres are indicated with orange spheres, the middle of each HIRs with blue spheres, and PF3D7_1240600 (active *var* gene) with green spheres. (J) Highlight of Chr13 (cyan) and Chr14 (yellow) in 3D models. Spheres represent the middle position of HIRs. Gray arrows represent the distance between HIRs of Chr13 and Chr14. (K) Changes in Euclidean distance of HIR-to-HIR located in intra and inter chromosomes and centromere-to-centromere between wild-type and *PfSET2*-KO samples. (L) Box plots of Euclidean distance between HIRs located at Chr13 and Chr14.

Our data showed that high levels of H3K36me3 and H3K9me3 were enriched at HIRs. It has been reported that PfHP1, which can bind to H3K9me3, plays an important role in regulating heterochromatin structure and depletion of PfHP1 leads to dramatic decrease of chromatin interactions in heterochromatin cluster (19–21). PfSET2 is also an epigenetic regulator to control H3K36me3 level on *var* genes, and knockout of *PfSET2* results in activation of all *var* genes (10). Because PfHP1 and H3K9me3 are well studied for their role in maintaining heterochromatin structure, we asked whether PfSET2 and H3K36me3 have similar function in regulating chromatin structure. Therefore, we performed Hi-C experiment in *PfSET2*-knockout sample at ring stage and compared the data with wild-type sample. By calling the HIRs in *PfSET2*-KO sample, we also identified HIRs that displayed similar size distribution and most of them overlapped with HIRs identified in wild-type sample (Fig. S3). Then, we compared the interaction frequency between wild-type and *PfSET2*-KO samples within HIRs. Surprisingly, deletion of *PfSET2* resulted in a marked reduction of both intrachromosomal and interchromosomal interactions for HIRs (Fig. 2A to 2C). Specifically, 65.84% interchromosomal and 37.93% intrachromosomal interactions between HIRs were decreased whereas the rest of HIR interactions were stable or weakly increased (Fig. 2D to 2G). Additionally, deletion of *PfSET2* showed no significant changes for non-HIRs regions with the median fold change ratio closed to zero (Fig. 2H).

To get a global view of chromatin organization in P. falciparum, we generated the 3D genome model inferred by our Hi-C data. Consistent with previous 3D modeling in P. falciparum (22–24), our 3D modeling showed that HIRs and centromeres were organized into two condense centers that located on the opposite sides of the nucleus, suggesting that HIRs and centromeres contribute to organized 3D structure of the P. falciparum genome (Fig. 2I; Fig. S4). When comparing the 3D model of the wild-type sample with *PfSET2*-KO, the cluster of HIRs in *PfSET2*-KO became significantly dispersed whereas centromeres cluster did not show marked change (Fig. 2I). To quantify such change by calculating the 3D distance between pairs of HIRs loci, 378 (66.67%) interchromosomal HIR pairs increased in 3D distance after deletion of *PfSET2*, while no significant change was found on intra-HIR pairs (Fig. 2K; Fig. S5A). In contrast, cluster of centromeres remained unchanged and still located on the opposite to HIR cluster, indicating that the loss of *PfSET2* only disrupted the interaction strength for HIR cluster (Fig. 2I and K; Fig. S4). When calculating the 3D distance between two individual chromosomes, chromosome 13 and chromosome 14, the *PfSET2*-KO sample showed a marked longer distance than the wild-type (Fig. 2J and L). In genome wide, the 3D distance between different chromosomes displayed modest increases after deletion of *PfSET2* (Fig. S5B). These results indicate that deletion of *PfSET2* leads to diminished interaction strength of HIRs for maintaining the stability of the repressive center.

### Reorganization of epigenetic modification and 3D genome by deletion of *PfSET2*.

**FIG 3:**
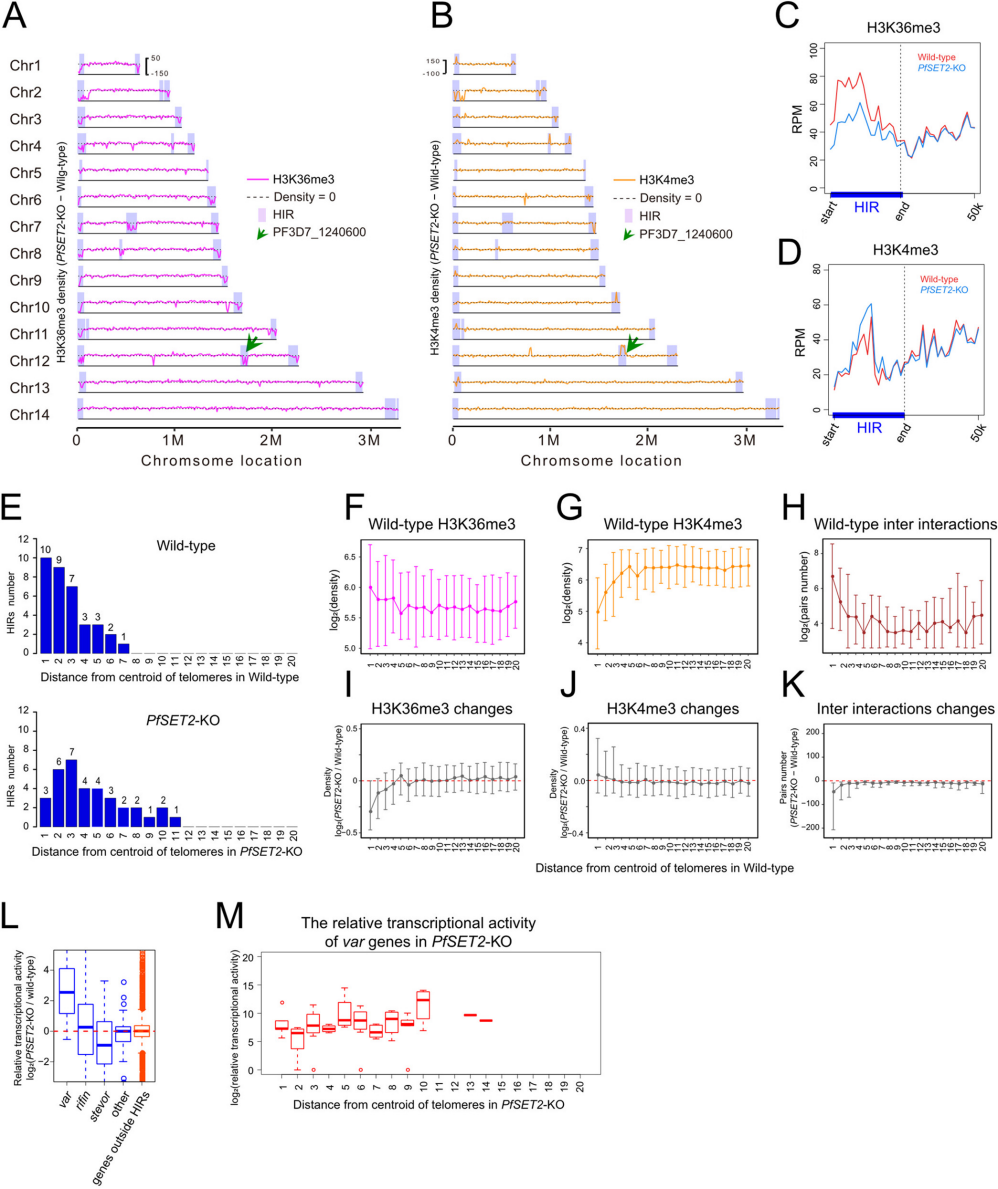
Reorganization of epigenetic modification and 3D genome by deletion of *PfSET2*. (A) and (B) Genome-wide changes of H3K36me3 (magenta) and H3K4me3 (orange) distribution at 10 kb resolution comparing *PfSET2*-KO with wild-type samples. HIRs are indicated with shadows. (C) and (D) Distribution of H3K36me3 and H3K4me3 ChIP-seq average signal at HIRs and 50 kb flanking regions in wide-type (red) and *PfSET2*-KO (blue) samples. (E) Relation between HIRs and distance from the centroid of the telomeres in wide-type and *PfSET2*-KO samples. Genome is divided into 20 bins according to increasing distance from the centroid of the telomeres. For each bin, the number of HIRs is counted and plotted above the bar. (F), (G) and (H) ChIP-seq signal of H3K36me3, H3K4me3 and frequency of interchromosomal interactions in regions with increasing distance from centroid of the telomeres in wild-type sample. For each bin, the median value of signal and total contacts frequency at each gene promoter regions are plotted. Error bars denote the first and third quartiles of signals in each bin. (I), (J) and (K) As in (F), but for changes of H3K36me3, H3K4me3 signal and interchromosomal interactions frequency between wide-type and *PfSET2*-KO samples. (L) The relative transcriptional activity changes for *var*, *rifin*, *stevor*, and other genes in HIRs (blue) and genes outside HIRs (orange). (M) Relation between the relative transcriptional activity of *var* genes in *PfSET2*-KO sample and distance from the centroid of the telomeres.

To understand how PfSET2 regulates HIRs and 3D genome organization, we further examined how H3K36me3 and other histone modifications change at HIRs in *PfSET2*-KO sample. H3K36me3, H3K9me3, and H3K4me3 ChIP-seq signal were profiled in wild-type and *PfSET2*-KO samples. Compared with wild-type data, occupancy of H3K36me3 was significantly decreased at HIRs regions (Fig. 3A and C). Interestingly, although chromatin interactions were markedly reduced at HIRs, the heterochromatin marker H3K9me3 has no significant change at most of HIRs. Previous works have shown that heterochromatin protein 1 (PfHP1) is the major factor to maintain the repressive center of heterochromatin, and depletion of PfHP1 led to a dramatic change in heterochromatin structure and particular loss of high-frequency interactions between virulence genes (20, 23). To investigate whether PfHP1 is involved in the reorganization of the 3D genome after deletion of *PfSET2*, we performed IFA and ChIP-seq assays of PfHP1 in wild-type and *PfSET2*-KO samples. Our IFA results showed no significant difference in the number of PfHP1 clusters in the nucleus between the two samples (Fig. S7A and B). Consistent with IFA results, the genome-wide distributions of PfHP1 binding were also similar in wild-type and *PfSET2*-KO samples (Fig. S7C and D). These results suggest that loss of chromatin interactions at HIRs is associated with the change of H3K36me3 and independent of H3K9me3 and PfHP1 binding in *PfSET2*-KO sample (Fig. S6A and B). When checking active histone mark H3K4me3, we found that about half of HIRs showed a marked increase of H3K4me3 in *PfSET2*-KO sample, indicating that these HIRs become more active in the absence of PfSET2 (Fig. 3B and D).

For a comprehensive view of how 3D genome architecture and epigenetic modifications change after *PfSET2* deletion, we binned the genome into 20 quantiles based on their increasing distance from the centroid of all telomeres and compared several chromatin features between wild-type and *PfSET2*-KO samples. In the wild-type sample, most of HIRs were located in the first three bins closest to the centroid of the telomeres that represented the repressive heterochromatin zone (Fig. 3E). In this zone, repressive chromatin marks H3K36me3 and H3K9me3, were highly enriched, whereas H3K4me3 occupancy was depleted compared with the rest of regions in the genome (Fig. 3F and G; Fig. S6C). In addition, this zone showed a high frequency of interchromosomal interactions (Fig. 3H). In the *PfSET2*-KO sample, the distribution of HIRs was not restricted in the first three bins closest to the centroid of the telomeres and spread to areas away from the telomere centroid, indicating that the HIRs center was dispersed in space (Fig. 3E). As expected, the loss of *PfSET2* resulted in a marked decrease of H3K36me3 and a weak increase of H3K4me3 in the repressive zone (Fig. 3I and J). Interchromosomal interactions in this zone were also significantly reduced (Fig. 3K). To study the relationship between transcriptional activity and genome organization, we calculated the average transcriptional activity in 20 quantiles using published data sets (10). We then plotted these values against their distance from the centroid of telomeres and the colored 20 quantiles in 3D models based on these values for wild-type and *PfSET2*-KO parasites Consistent with previous studies, wild-type parasites showed the lowest transcriptional activity in the closest bin to the centroid of the telomeres, a gradient increasing in transcriptional activity in the next seven bins and comparable transcriptional activity in the remaining bins (Fig. S8) (22, 24). Compared with wild-type parasites, *PfSET2*-KO parasites showed a strong increase in transcriptional activity in the bin closest to the centroid of the telomeres and the transcriptional activity comparable with the activated bins, suggesting the decrease of the repressive chromatin state at HIRs (Fig. S8). Notably, the 3D space redistribution of HIRs in *PfSET2*-KO sample was correlated with transcriptional activation of *var* genes which showed a gradient increasing from telomere centroid (Fig. 3M). Again, these results confirmed the major role of PfSET2 in regulating chromatin organization of the repressive zone.

### Interactions associated with *var* genes is controlled by PfSET2.

**FIG 4:**
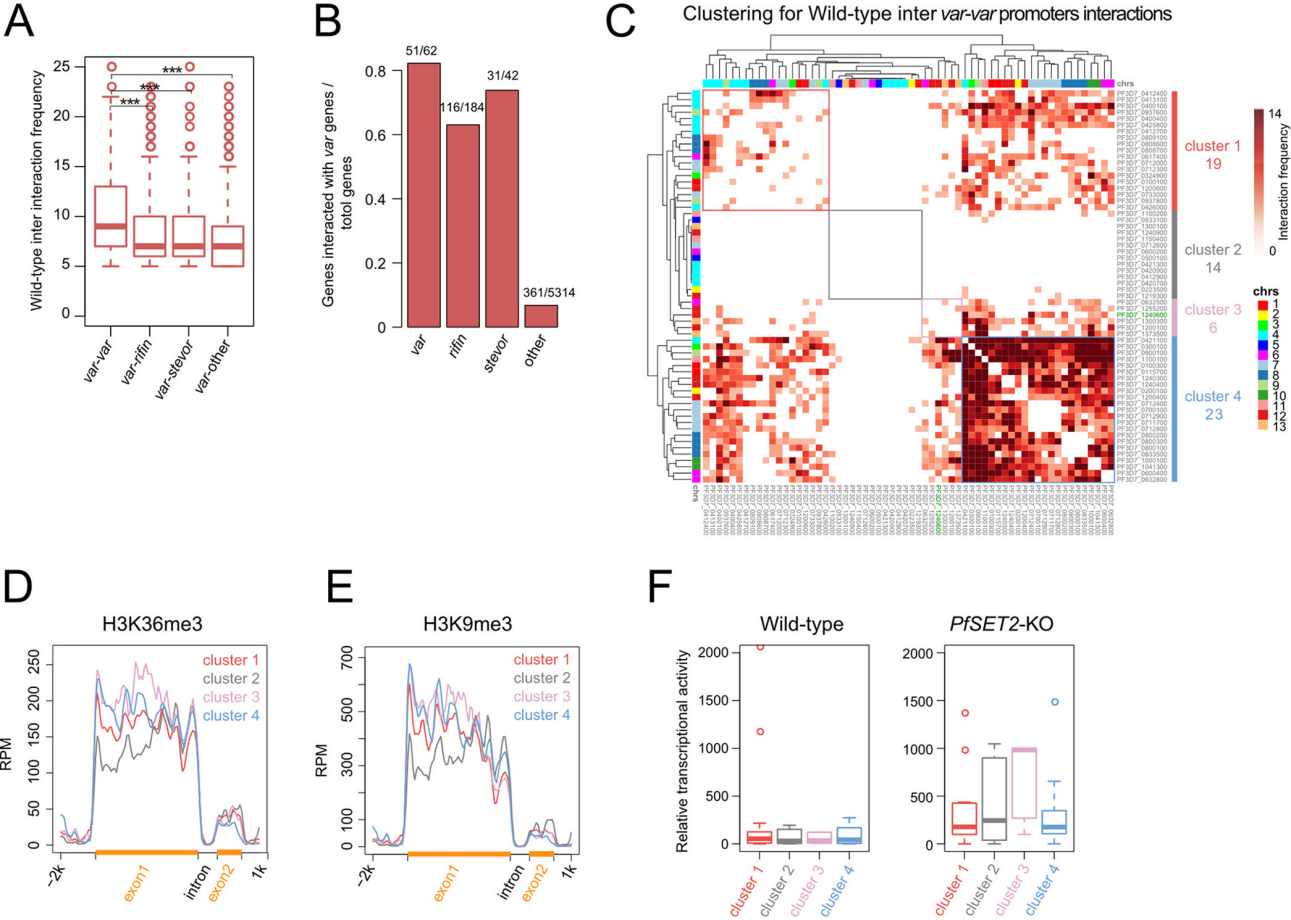
Interactions associated with *var* genes is controlled by *PfSET2*. (A) *Var* gene promoters associated interactions are classified into *var*-to-*var*, *var*-to-*rifin*, *var*-to-*stevor*, *var*-to-other in wild-type sample. (B) The percentage of genes that are interacted with *var* gene promoters in each genes family for interaction groups in (A). (C) K-means clustering analysis of *var*-to-*var* promoter interactions based on contact frequency in wild-type sample. Chromosomes are indicated with different colors. (D) and (E) Distribution of H3K36me3 and H3K9me3 ChIP-seq average signal along genes in groups described in (C). (F) Box plots show genes relative transcriptional activity of each group in wild-type (right) and *PfSET2*-KO (left) samples (the uniquely expressed var gene in wild-type sample is not included).

To understand how 3D genome organization contributed to transcription, especially the silencing of *var* genes within HIRs, we investigated chromatin interactions associated with *var* genes. Specifically, chromatin interactions associated with *var* gene promoters were classified into four groups by the type of genes of their interacting promoter regions. In both wild-type and *PfSET2*-KO samples, promoter regions of *var* genes showed higher interaction frequency with promoters of other *var* genes than with promoters of other gene families, such as *rifin*, *stevor* (Fig. 4A; Fig. S9B). In addition, about 82% (51 of total 62) of the *var* genes promoters formed *var-var* interactions, while 63% (116 of total 184) of *rifin* genes and 74% (31 of total 42) of *stevor* genes were involved in *var-rifin* interactions and *var-stevor* interactions, respectively. The proportion of genes other than these three gene families that interacted with *var* genes were much lower, indicating the three gene families were the major interaction partners of *var* genes (Fig. 4B). Interestingly, in *PfSET2*-KO sample, the numbers of genes promoter interactions of the three families that interacted with *var* gene promoters were all marked decreased compared with the wild-type sample, suggesting loss of *PfSET2* impairs the interactions between *var*, *rifin*, and *stevor* genes (Fig. S9A and B). To further investigate the patterns of *var-var* interactions, we performed hierarchical clustering, based on promoter regions interaction frequency between any pair of two *var* genes, and identified four clusters in wild-type sample (Fig. 4C). Hierarchical clustering analysis revealed that: (i) 23 *var* gene promoters in cluster 4 strongly interacted with each other; (ii) 14 *var* gene promoters in cluster 2 did not interact with any *var* genes; (iii) cluster 3, which contained the expressed *var* gene (PF3D7_1240600), showed modest interactions with cluster 4; (iv) cluster 1 had only a few interactions within the cluster and weakly interacted with cluster 4.

To further explore the underlying mechanism of *var* genes promoters clustering, we first examined the histone marks enrichment of the four clusters. Clusters 1, 3, and 4 that interacted with each other exhibited higher enrichment for H3K36me3 and H3K9me3 at gene promoter regions, while cluster 2 which did not interact with other clusters showed lower enrichment (Fig. 4D and E), indicating that H3K36me3 and H3K9me3 occupancy might correlate with *var* promoter interactions. Furthermore, we checked the expression pattern of *var* genes in the four clusters and found that the *var* genes in cluster 3, which contained the uniquely expressed *var* gene (PF3D7_1240600) in wild-type sample, showed much higher transcriptional activity than other clusters after deletion of *PfSET2* (Fig. 4F). Taken together, these results suggest that PfSET2 regulates chromatin interactions associated with *var* genes promoter and the different interaction patterns of *var* genes correlate with repressive marks of H3K36me3 and H3K9me3 as well as the activation potential of *var* genes in *PfSET2-*KO sample.

## DISCUSSION

**FIG 5:**
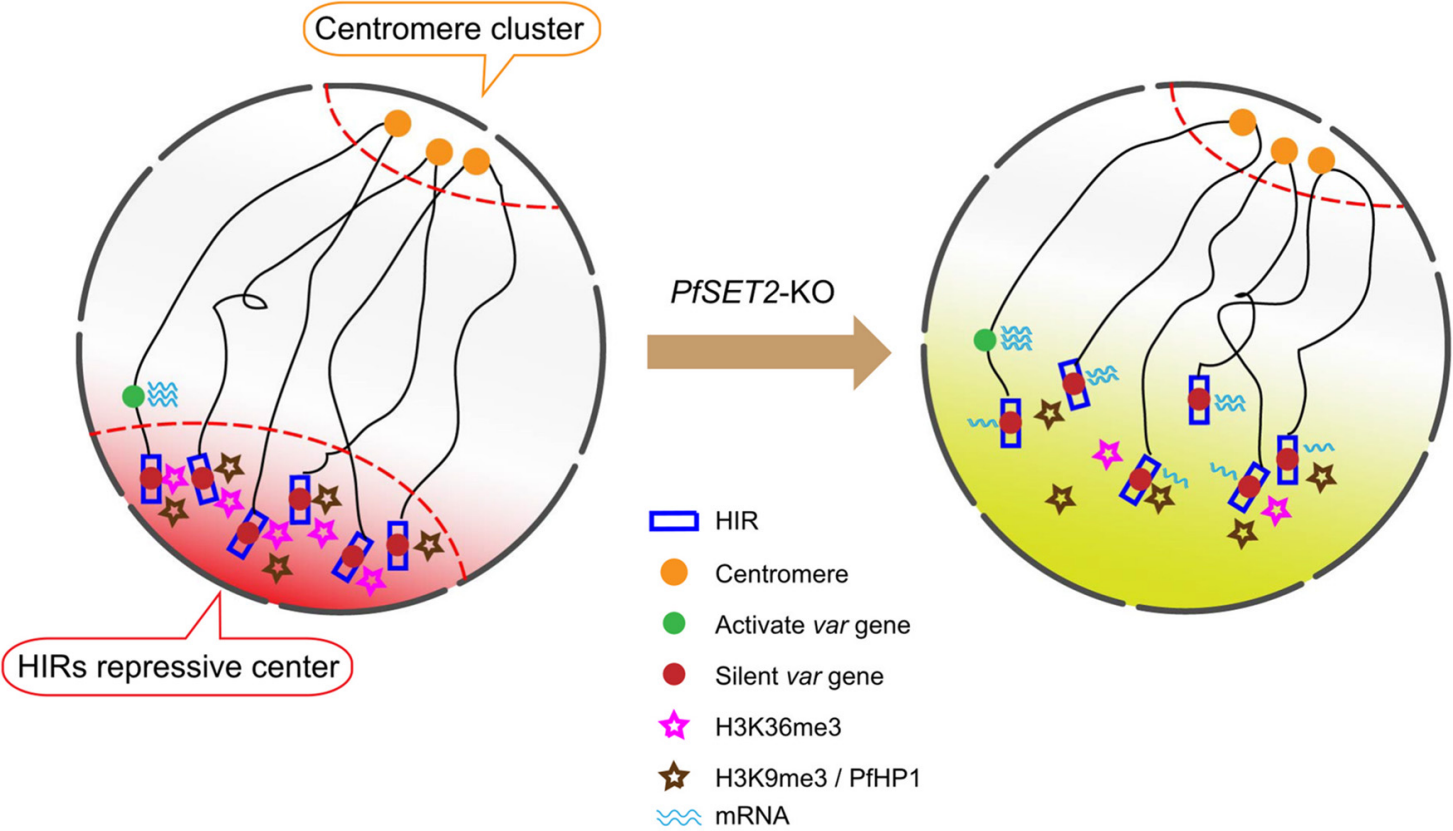
A model of *PfSET2* in regulating 3D genome organization and *var* gene transcription activity. HIRs (blue rectangles) repressive center and centromeres (orange circles) cluster localized on opposite sides at the nucleus periphery. HIRs are located within the repressive center in wild-type parasites (red shadow, left) while become active state in *PfSET2*-KO parasites (yellow shadow, right). Active *var* gene (green circle) localizes outside HIRs repressive center. Silent *var* genes (red circles) are actively transcribed (mRNA, cyan wavy lines) in *PfSET2*-KO parasites (right).

In this study, we propose a model of how PfSET2 regulates transcription of *var* gene family via mediating the epigenome and high-order structure of genome organization in P. falciparum (Fig. 5). In wild-type parasite, clustering of HIRs forms the repressive center enriched with heterochromatin marks H3K36me3, H3K9me3, and PfHP1. This repressive center is located opposite to euchromatin centromere cluster at nuclear periphery with distinct chromatin environments (Fig. 5, left). In *PfSET2*-KO parasite, the loss of *PfSET2* leads to decrease of H3K36me3 and reduced interaction strength among HIRs cluster that markedly change the heterochromatin status (Fig. 5, right). Consequently, the original silent *var* genes are activated. Therefore, our study demonstrates the essential role of PfSET2 in regulating the heterochromatin organization associated with the silence of *var* genes in P. falciparum.

Genome organization of P. falciparum has important functions in regulating gene expression (22–24, 26, 28). In mammals, chromosomes are partitioned into mega-base-sized topological associated domains (TADs) and compartment structure that can coordinately regulate epigenetic state and gene expression (31, 35–39). However, only domain-like structures around VRSM genes cluster have been reported in P. falciparum at intrachromosomal level (22, 23). In our study, we precisely identify the HIRs that are interacted strongly with each other in 3D space both of intra- and interchromosomal. We find that HIRs also exhibit domain-wide features of heterochromatin histone marks (H3K36me3 and H3K9me3) and largely overlap VRSM genes loci. Previous studies have also demonstrated that nucleus is compartmentalized into at least three distinct areas in which nuclear proteins PfSET2 (encoded by MAL13P1.122) and repressive histone marks H3K36me3 and H3K9me3 are located to the nuclear periphery forming a third compartment that contributes to maintaining the heterochromatin environment to silence virulence gene families (10, 19, 20, 40). Changes of genome organization and histone modifications in HIRs are well correlated with the change of transcriptional activity of *var* gene family, which may be important for studying immune evasion mechanism to avoid the host antibody response.

Previous works have reported that clustering of telomeres forms the repressive center located at nuclear periphery opposite to the clustering of centromeres in P. falciparum (23, 24). Consistent with these reports, our HIRs are enriched with repressive histone marks H3K36me3 and H3K9me3. We find that deletion of *PfSET2* leads to marked decrease of H3K36me3 at *var* genes loci and the interaction strength between HIRs. Interestingly, the interaction hub formed by HIRs is not completely lost in *PfSET2* knockout sample. As the H3K9me3 level at *var* gene loci is not affected by deletion of *PfSET2*, it is possible that PfHP1 maintains the heterochromatin organization in the absence of PfSET2 although the transcription activities in these regions are largely increased. In addition, deletion of *PfSET2* did not affect the binding of PfHP1 to chromatin and the location of PfHP1 scattered around the nucleus, suggesting that PfSET2 plays a role in maintaining the status of repressive center independent of PfHP1 (Fig. S7C and D). Thus, we propose that PfSET2 and PfHP1 are both the major forces regulating 3D structure of heterochromatin by controlling repressive histone marks H3K36me3 and H3K9me3, respectively. In the future, it would be interesting to examine whether the 3D structure of the HIR hub is completely lost if the both factors are deleted.

HIRs show sharp boundaries where distinct chromatin interaction frequency and enrichment of repressive histone marks (H3K36me3 and H3K9me3) are observed around HIRs boundaries. We also find that intra- and interaction frequency and H3K36me3 enrichment are changed within HIRs and delimited by boundaries. In mammals, CCCTC-binding factor (CTCF) acts as an insulator protein in the genomic organization and gene regulation (41–45). In P. falciparum, homologs of CTCF have not been identified (24, 45). Therefore, it remains to determine which factor could be involved in the insulation function.

In addition to PfHP1 and PfSET2, several other factors have been reported to be involved in 3D genome organization of malaria parasite. The high-mobility-group protein PfHMGB1 is recruited mainly to centromeric regions to maintain centromere/telomere-dependent genome organization (46). The loss of PfHMGB1 results in markedly reduced interaction frequency among centromere clusters and silencing of mutually exclusive *var* gene. Recently, it is reported that a novel chromatin-associated DNA-binding protein Homeo Domain Protein 1 (HDP1) is tightly associated with genome organization. Loss of HDP1 leads to significant reduction in interaction frequency within telomeric clustering and dysregulation of gene expression in early gametocytes (47). Chromatin structure is also subjected to dynamic regulation during the parasite life cycle which contributes to transcriptional regulation of specific gene families (23). In the future, it will be of great interest to identify novel factors that are involved in regulating 3D genome architecture during the parasite life cycle.

Based on Hi-C data, 3D modeling of P. falciparum at 10 kb resolution reveals that the HIRs are organized into a single large cluster. This result is in agreement with previous studies that IFA on H3K9me3 or IFA on PfHP1 show a single focus (8, 20, 23, 48, 49). By further examining the interaction pattern at *var* genes levels, our results show that the promoters of *var* genes can be classified into four clusters, with different interaction patterns and activation potential in *PfSET2*-KO sample. Interestingly, the singular expressed *var* gene in wild type parasite is in group 3 (Fig. 4C) and other *var* genes in the same group show highly transcriptional activation level after deletion of *PfSET2* (Fig. 4F).

Compared with the single cell analysis FISH or IFA technologies, Hi-C can detect genome-wide chromatin interactions at much higher resolution (22–24, 30, 46, 47). However, Hi-C data are usually produced from bulk of cells and represent the average contact frequencies in a cell population. FISH and IFA are single cell analysis for examining the spatial localization of genomic DNA and proteins although the resolution and through-put are much lower than Hi-C assay (16, 17, 20, 21, 40). Therefore, it is important to verify the results based on Hi-C data by single cell FISH or IFA assay.

In summary, this study has provided a fundamental mechanism of how epigenetic factor PfSET2 regulates the 3D organization of heterochromatin, which has key function in controlling the transcription activities of *var* genes family in P. falciparum. It is clear that epigenetic modifications and genome organization can act as the switch for controlling the *var* genes expression. Studying the factors that could affect the chromatin structure and transcription of *var* genes may contribute to identification of new targets of malaria intervention strategies.

## MATERIALS AND METHODS

### Parasite culture and transfection.

P. falciparum was cultured according to a standard protocol (50). All parasites were cultured in an atmosphere consisting of 5% CO_2_, 5% O_2_, and 90% N_2_. Highly synchronous cultures of ring-stage parasites were used for the study. For Hi-C study, parasites were cross-linked by 1% formaldehyde for 10 min at room temperature followed by addition of 0.125 M glycine to stop cross-linking. After the saponin lysis (0.15%), the supernatant was removed and the samples were stocked in −80°C after frozen with liquid nitrogen.

### ChIP-seq library preparation.

Crosslinked chromatin was sheared by sonication in a Q-Sonica for 10 min at 30-s intervals, power setting high, to a size of 200 to 300 bp. Chromatin samples were frozen and stored at −80°C. ChIP was performed as described previously (10). In brief, antibodies to HP1 were added to cross-linked samples of wild-type 3D7 and 3D7SET2Δ, and incubated at 4°C, followed by the addition of 10 mL A/G beads and further incubation for 2 h. After washing with buffers containing 100, 150, and 250 mM NaCl, immunoprecipitated DNA was eluted and purified using PCR purification columns (Qiagen). The ChIP-seq library construction was processed with QIAseq ultralow input library kit. We used Illumina HiSeq X 10 to perform the pair-end sequencing.

### Hi-C library preparation.

In situ Hi-C was performed as previously described with moderate modifications (30, 31). Briefly, fixed parasites were lysed in 5 mL ice-cold Hi-C lysis buffer (10 mM Tris-HCl pH 7.5, 10 mM NaCl, 0.2% NP-40, 1× protease inhibitors) and rotated at 4°C for 1 h. Pelleted nuclei were resuspended with 300 μL H2O, 44 μL of 10×NE Buffer 2, and 38 μL of 1% SDS and incubated at 65°C for 10 min. Then, 44 μL of 10% Triton X-100 were added and samples were rotated at 37°C for 60 min to quench the SDS. To fragment DNA, 350 U endonucleases DpnII (NEB, R0543M) was added to the samples and samples were incubated at 37°C overnight. Digested DNA was filled in the restriction fragment overhangs with Klenow (NEB, M0210L) and the DNA ends was marked with biotin. Chromatin was then ligated with T4 DNA ligase (Thermo Fisher Scientific, EL0013) for 4 h at 16°C. DNAs were sonicated to reduce their size to 300 to 500 bp and immobilized on Dynabeads M-280 Streptavidin (Invitrogen,11205D).The process of ends repair, A-tailing, and adaptor ligation referred to ChIP-seq method (51), the Hi-C library was amplified for 12 to 13 cycles, and the PCR product was loaded on 1% agarose and size selected the 300 to 600 bp band for sequencing.

### ChIP-seq data analysis.

ChIP-seq reads were mapped to the P. falciparum *3D7* reference genome (PlasmoDB release 43) by using bowtie v.1.1.1 with default parameters. Duplicate reads were excluded and kept only one read for each genomic site. ChIP-seq signals were quantile normalized to remove the bias across the samples by using haystack_bio v.0.5.5 (https://github.com/pinellolab/haystack_bio). BedGraph files were normalized for total mapped read counts and signal was calculated as per million per kilo-base (RPM). The normalized reads density bigwig tracks were used for visualization with Integrative Genomics Viewer (IGV) (52). To generate the ChIP-seq signal distribution for interested regions, we calculated the average ChIP-seq signal across these regions.

### Hi-C data processing.

Hi-C libraries were sequenced on the Illumina NovaSeq 6000 system to obtain 2*150 bp paired-end reads. Raw sequencing reads were aligned to P. falciparum *3D7* reference genome (PlasmoDB release 26), processed, and ICE normalized using HiCPro package (version 2.11.4) (53, 54). Valid read pairs of biological replicates were pooled. To eliminate the possible effects on data analyses of variable sequencing depths, we pooled valid read pairs of biological replicates and then normalized each sample to contain the same number of valid read pairs for downstream analyses. For examination of global contact patterns, valid read pairs were then aggregated into contact matrix at 10 kb resolution and normalized using ICE method to correct for experimental and technical biases. We used GotHiC at 2 kb resolution to identify long-range chromatin interactions (55). Interactions with pairs number ≥5 and *P*-value ≤ 0.1 were considered high-confidence interactions. Additionally, intrachromosomal interactions that linear distance ≤ 10 kb were filtered out.

### HIRs calling.

To systematically identify HIRs at whole genome, we first called high level intra- and interchromosomal interacting regions separately. High-level intrachromosomal interacting regions calling consisted of four steps: (i) for each chromosome, removing contacts along the diagonal regions that were located 20% length of each chromosome around diagonal; (ii) keeping the bins in which contact frequency ranked on the top 3% of each contact matrix; (iii) keeping the continuous bins and removing the separated bins on contact matrix; (iv) mapping bins on contact matrix to linear genome and getting high level interacting regions. High-level interchromosomal interacting regions calling contained steps (ii), (iii), and (iv) described above in high-level intrachromosomal calling. We then considered overlap regions between high-level intrachromosomal interacting regions as HIRs.

### 3D modeling and visualization.

A consensus 3D genome structure of each samples were inferred using the Pastis (v0.1) (https://github.com/hiclib/pastis) method with a Poisson model (PM2) (56). The 10 kb contact matrixes were used to construct the 3D models. 3D models were visualized using Jmol (jmol.sourceforge.net/). PDB files were available in supplementary.

### Correlation between genomic features (HIRs location, epigenome, interactions, *var* gene expression) and 3D models.

All the 10 kb genomic bins were sorted by increasing distance to the centroid of telomeres and combined into 20 equal width quantiles (*x* axis). For HIRs location, we counted the numbers of HIRs in each quantile (*x* axis). For the ChIP-seq density of H3K36me3, H3K9me3, and H3K4me3, we calculated the signal at the gene promoter regions (tss-1k) and plotted the range of signals in each quantile (*x* axis). For chromatin interactions, we summed the contact frequency of interactions located at genes promoter regions of each genes and plotted the range of values in each quantile. For *var* genes expression, we plotted log_2_(relative transcriptional activity) of *var* genes in each quantile in *PfSET2*-KO sample.

### Data and code availability.

HIRs Calling is an open source collaborative initiative available in the GitHub repository (https://github.com/XuanCao-CX/HIRsCalling). Hi-C sequencing data and pfHP1 ChIP-seq data of this study have been deposited in the NCBI Gene Expression Omnibus (GEO) under accession numbers GSE169028 and GSE193761. We used microarray data from NCBI Gene Expression Omnibus (GEO) under accession number GSE47349. We used H3K36me3, H3K9me3, H3K4me3, H4K20me3, and H3K36me3 ChIP-seq raw data are available from Sequence Read Archive (SRA) database under accession number SRP022761.
