# Supplementary material for: PfSET2 Is Involved in Genome Organization of Var Gene Family in Plasmodium falciparum
Source: Microbiol Spectr. 2023 Jan 5;11(1):e03891-22. doi: 10.1128/spectrum.03891-22 (PMC9927267; doi:10.1128/spectrum.03891-22)
Supplement: Supplemental file 1 — Supplemental material. Download spectrum.03891-22-s0001.pdf, PDF file, 7.5 MB [file spectrum.03891-22-s0001.pdf]

Table S1. Genomic locations of HIRs in wild-type sample.

chr	start	end	name
Pf3D7_01_v3	20000	70000	HIRs_1
Pf3D7_01_v3	590000	640000	HIRs_2
Pf3D7_02_v3	0	70000	HIRs_3
Pf3D7_02_v3	840000	880000	HIRs_4
Pf3D7_02_v3	890000	947102	HIRs_5
Pf3D7_03_v3	0	80000	HIRs_6
Pf3D7_03_v3	1000000	1067971	HIRs_7
Pf3D7_04_v3	0	90000	HIRs_8
Pf3D7_04_v3	960000	990000	HIRs_9
Pf3D7_04_v3	1130000	1200000	HIRs_10
Pf3D7_05_v3	20000	50000	HIRs_11
Pf3D7_05_v3	1320000	1343557	HIRs_12
Pf3D7_06_v3	0	40000	HIRs_13
Pf3D7_06_v3	1330000	1418242	HIRs_14
Pf3D7_07_v3	0	60000	HIRs_15
Pf3D7_07_v3	500000	610000	HIRs_16
Pf3D7_07_v3	1380000	1440000	HIRs_17
Pf3D7_08_v3	0	60000	HIRs_18
Pf3D7_08_v3	430000	460000	HIRs_19
Pf3D7_08_v3	1390000	1472805	HIRs_20
Pf3D7_09_v3	20000	60000	HIRs_21
Pf3D7_09_v3	1480000	1510000	HIRs_22
Pf3D7_10_v3	0	70000	HIRs_23
Pf3D7_10_v3	1600000	1687656	HIRs_24
Pf3D7_11_v3	0	80000	HIRs_25
Pf3D7_11_v3	1980000	2038340	HIRs_26
Pf3D7_12_v3	0	70000	HIRs_27
Pf3D7_12_v3	1670000	1750000	HIRs_28
Pf3D7_12_v3	2160000	2260000	HIRs_29
Pf3D7_13_v3	20000	90000	HIRs_30
Pf3D7_13_v3	2850000	2920000	HIRs_31
Pf3D7_14_v3	0	60000	HIRs_32
Pf3D7_14_v3	3150000	3260000	HIRs_33
Pf3D7_14_v3	3270000	3291936	HIRs_34

Figure S1

A

Sample	Raw reads	Valid pairs	Unique valid pairs	Intra pairs 1kb-10kb	Intra pairs >10kb	Intra pairs
Wild-type rep1	70,636,397	33,391,556	28,179,913	2,531,750	10,149,966	15,498,197
Wild-type rep2	51,710,792	25,249,636	21,463,825	1,897,066	7,800,237	11,766,522
PfSET2-KO rep1	46,650,875	21,729,220	18,271,641	1,499,366	6,558,216	10,214,059
PfSET2-KO rep2	48,123,390	23,873,133	20,091,602	1,680,143	7,218,614	11,192,845

B

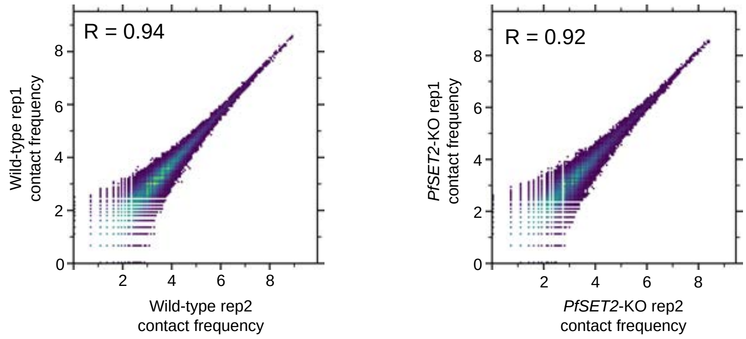

Figure S1. Summary of Hi-C sequencing data and correlation between replicates. (**A**) Numbers of sequence reads generated in Hi-C experiments and valid pairs. (**B**) Scatter plots showing correlation of Hi-C contact matrix at 10kb resolution between biological replicates using HiCexplorer.

Figure S2

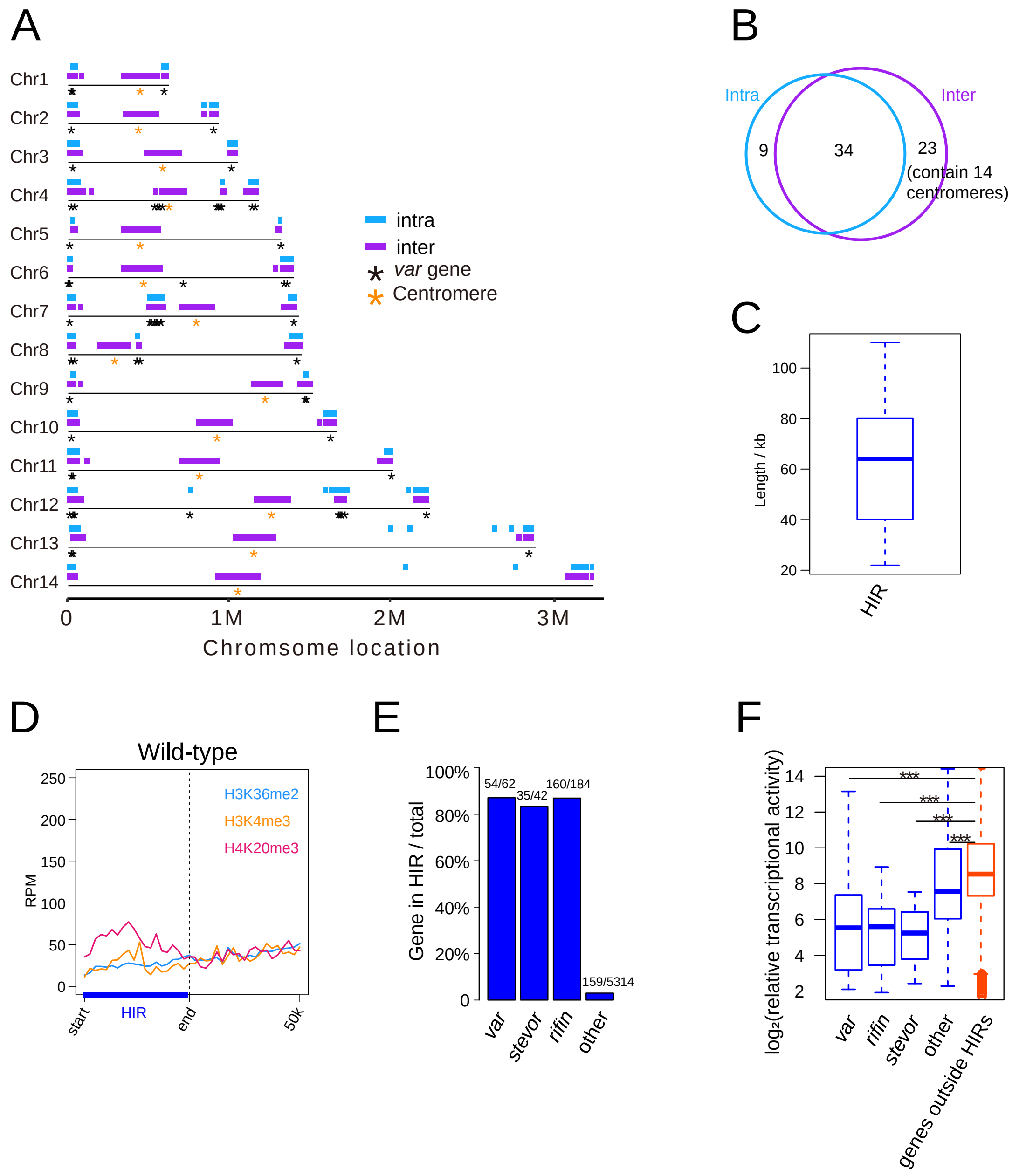

Figure S2. Genomic and epigenetic features of HIRs. (**A**) High level intra- (blue) and inter- (purple) chromosomal interaction regions. *Var* genes loci are indicated with black *, centromeres with yellow *. (**B**) Overlap between high level intra- and inter- chromosomal interaction regions. (**C**) Box plot of HIRs length. (**D**) Distribution of H3K4me3, H4K20me3 and H3K36me2 ChIP-seq average signal at HIRs. (**E**) Bar plot shows the percentage of *var*, *rifin*, *stevor* and other (the rest of the genes) genes located in HIRs in each gene families. Numbers above bars indicate the percentages per gene families. (**F**) Box plots of gene expression (log2 (relative transcriptional activity)) of *var, rifin, stevor* and other genes in HIRs (blue) and genes located outside HIRs (orange). All box plots depict the first and third quartiles as the lower and upper bounds of the box, with a thicker band inside the box showing the median value and whiskers representing 1.5× the interquartile range.

Figure S3

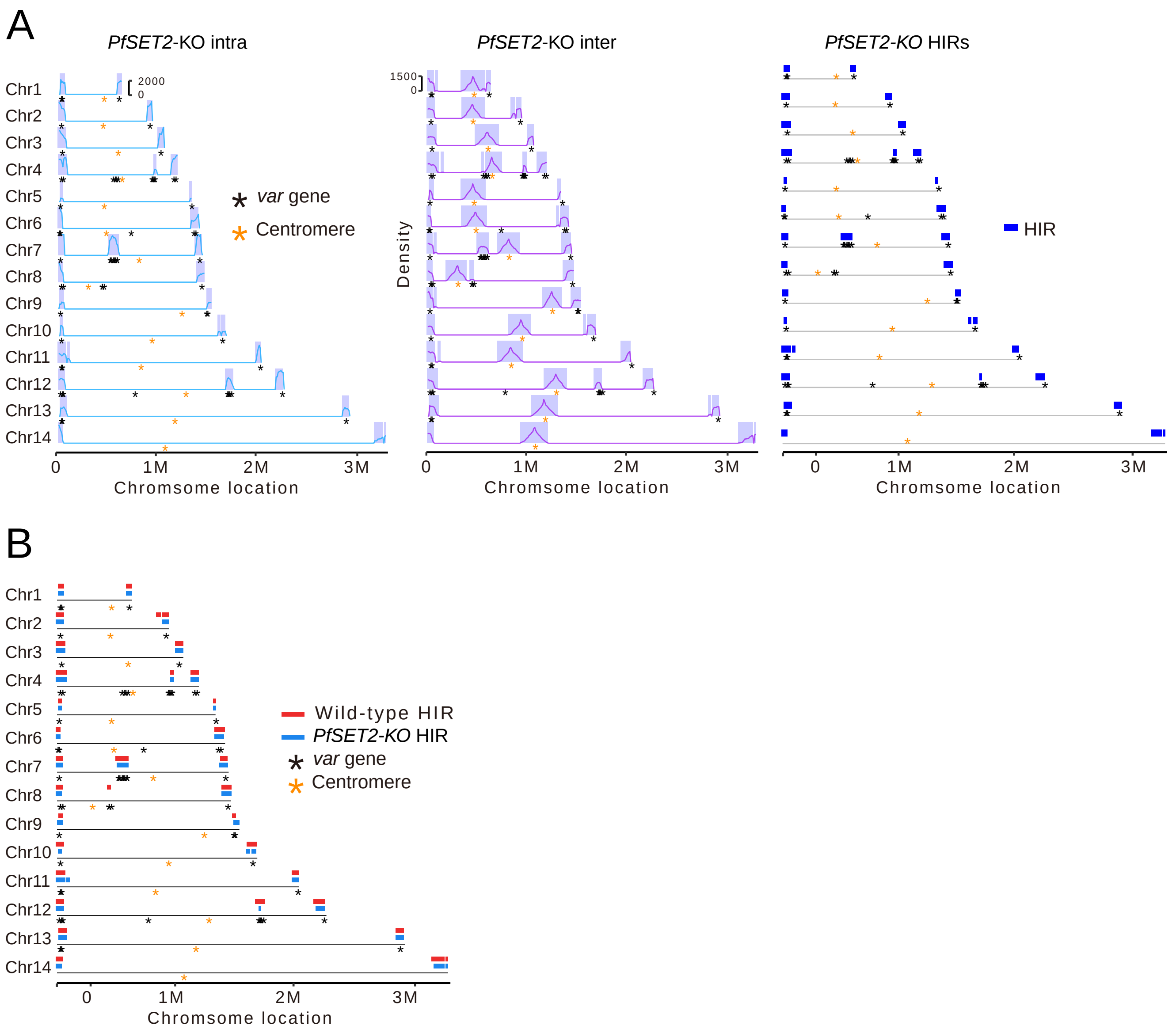

Figure S3. HIRs calling in *PfSET2*-KO sample. (**A**) Genome-wide locations of HIRs in *PfSET2*-KO sample. Left and middle: shadows indicate high level intra- and inter- chromosomal interaction regions, blue lines and purple lines are the contact frequency. Right: Genomic location of HIRs. *Var* genes loci are indicated with black *, centromeres with yellow *. (**B**) Genomic locations of HIRs in wild-type (red) and *PfSET2*-KO (blue) samples.

Figure S4

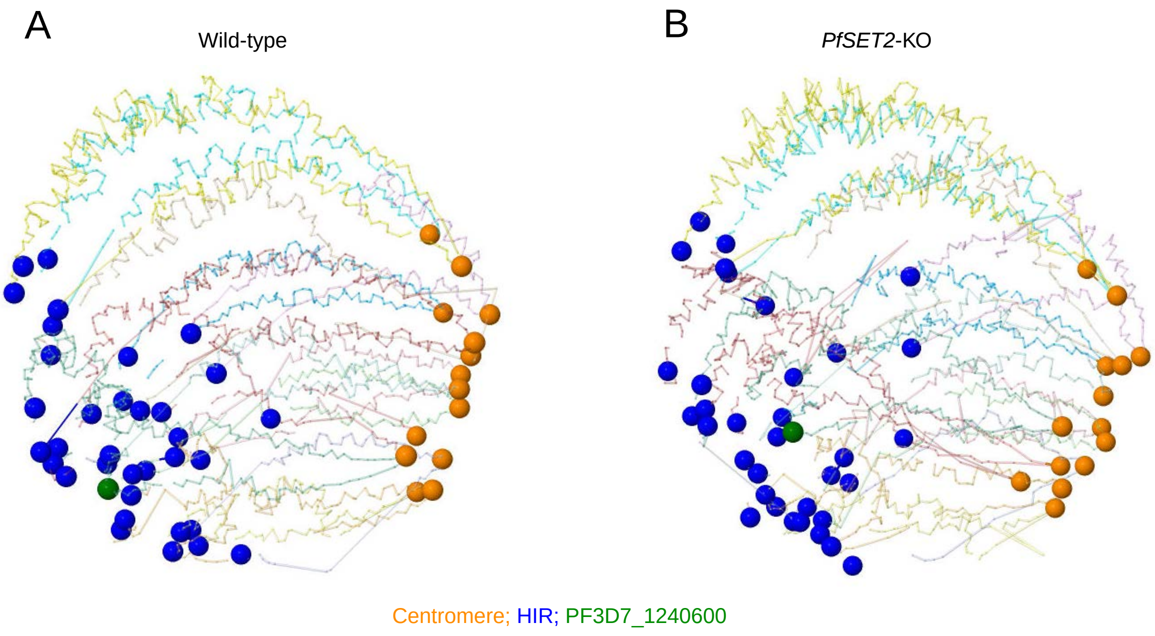

Figure S4. 3D models for wild-type (**A**) and *PfSET2*-KO (**B**) samples. Chromosomes are shown as transparent ribbons with different colors. Centromeres are indicated with orange spheres, middle of each HIRs with blue spheres, and PF3D7_1240600 (expressed *var* gene) with green spheres.

Figure S5

A

Euclidean distance log2(*PfSET2*-KO/wild-type)	HIR pairs of inter chr	HIR pairs of intra chr
Distance is farther	378 (66.67%)	12
Distance is closer	72 (12.70%)	8
No change	117 (20.63%)	8

B

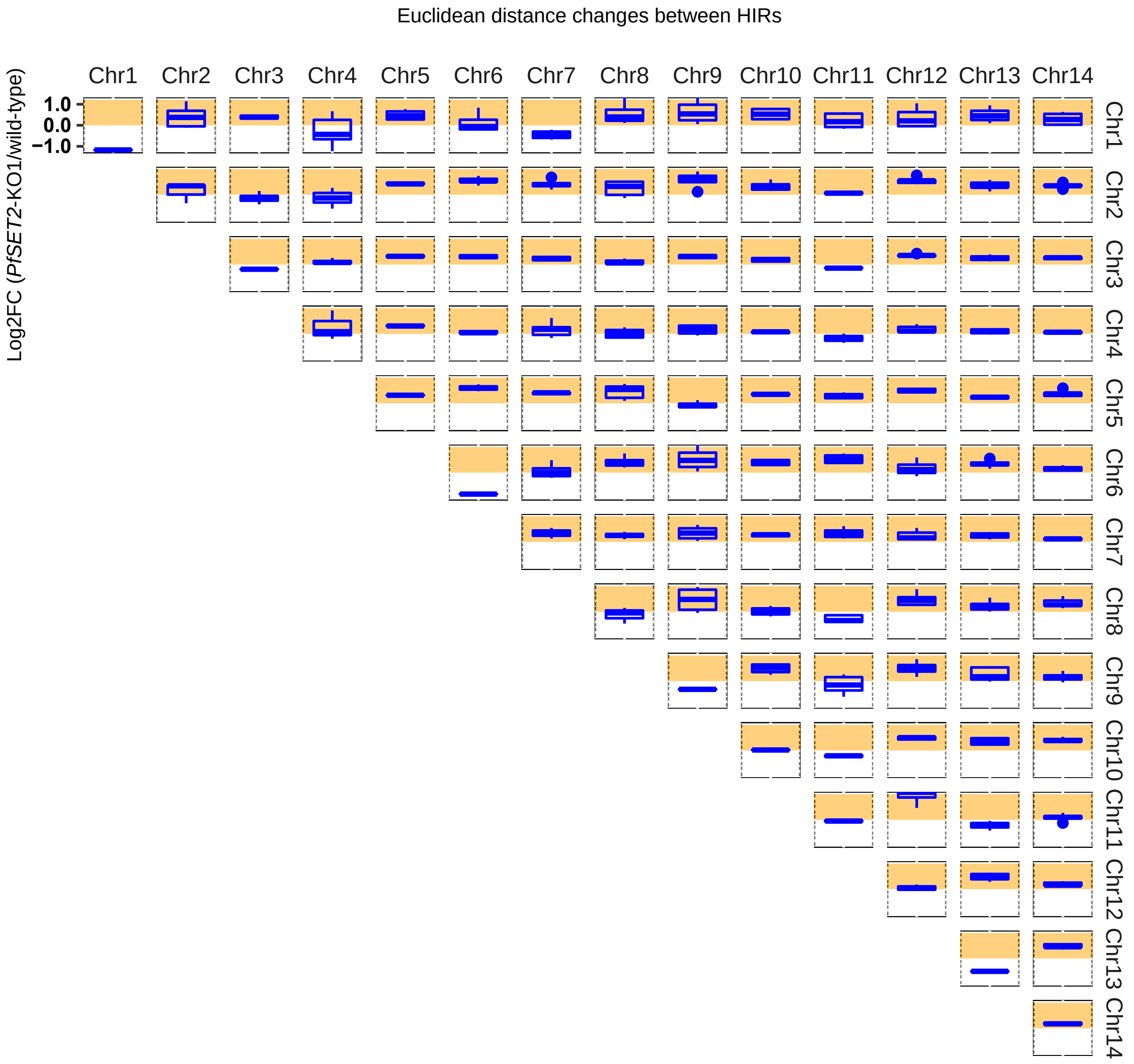

Figure S5. 3D distance changes. (**A**) Euclidean distance changes of HIR pairs in wild-type and *PfSET2*-KO parasites. Number of pairs that distance become farther and closer (|log2(*PfSET2*-KO/wild-type) | >= 1.1) are computed respectively. (**B**) Box plots showing euclidean distance changes of intra- and inter- chromosomal HIR pairs for each chromosome in wild-type and *PfSET2*-KO samples.

Figure S6

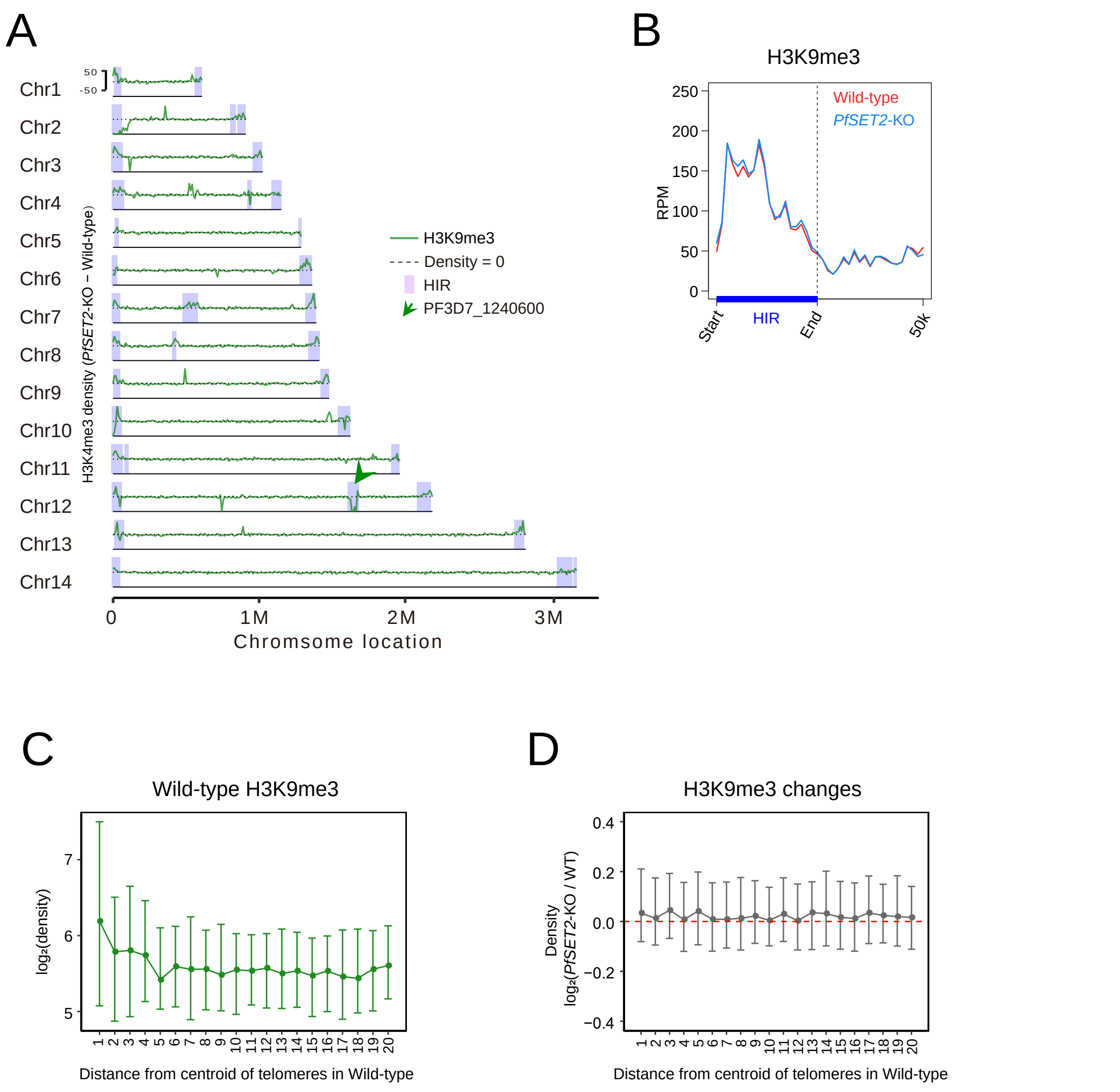

Figure S6. Relation between HIRs and H3K9me3 occupancy. (**A**) Distribution of H3K9me3 ChIP-seq average signal at HIRs and 50kb flanking regions in wild-type (red) and *PfSET2*-KO (blue) samples. (**B**) Genome-wide H3K9me3 ChIP-seq signal changes at 10kb resolution. HIRs are indicated with shadows. (**C**) H3K9me3 signal at gene promoter regions with increasing distance from centroid of the telomeres in wild-type sample. For each bin, the median value of H3K9me3 signal at genes promoter regions was plotted. Error bars denote the first and third quartiles of signals in each bin. (**D**) Changes of H3K9me3 ChIP-seq signal with increasing distance from centroid of the telomeres comparing *PfSET2*-KO with wild-type samples.

Figure S7

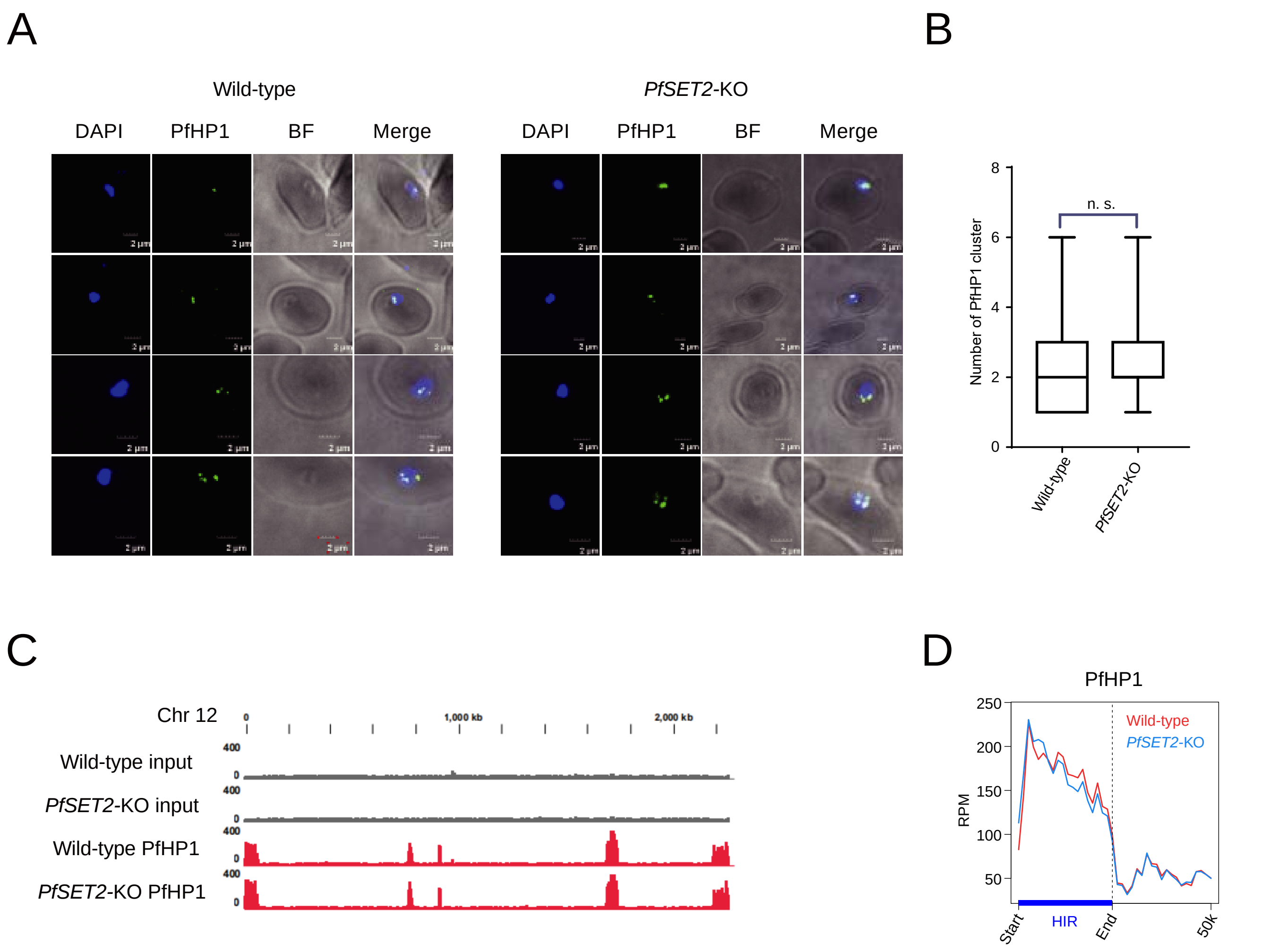

Figure S7. PfHP1 shows no significant change after deletion of *PfSET2*. (**A**) IFA assay of PfHP1 (rabbit anti-HP1 antibody, green) in wild-type and *PfSET2*-KO parasites at ring stage.Nuclear DNA was stained by DAPI (blue). Scale bar: 2μm.(**B**) The number of PfHP1 foci per nucleus was counted in about 200 ring stage parasites of wild-type and *PfSET2*-KO strain.The result is displayed in a box graph.(**C**) PfHP1 ChIP-seq signal distribution of Chr12 in wild-type and *PfSET2*-KO parasites. (**D**) ChIP-seq enrichment profile of PfHP1 at HIRs in wild-type (red) and *PfSET2*-KO (blue) parasites.

Figure S8

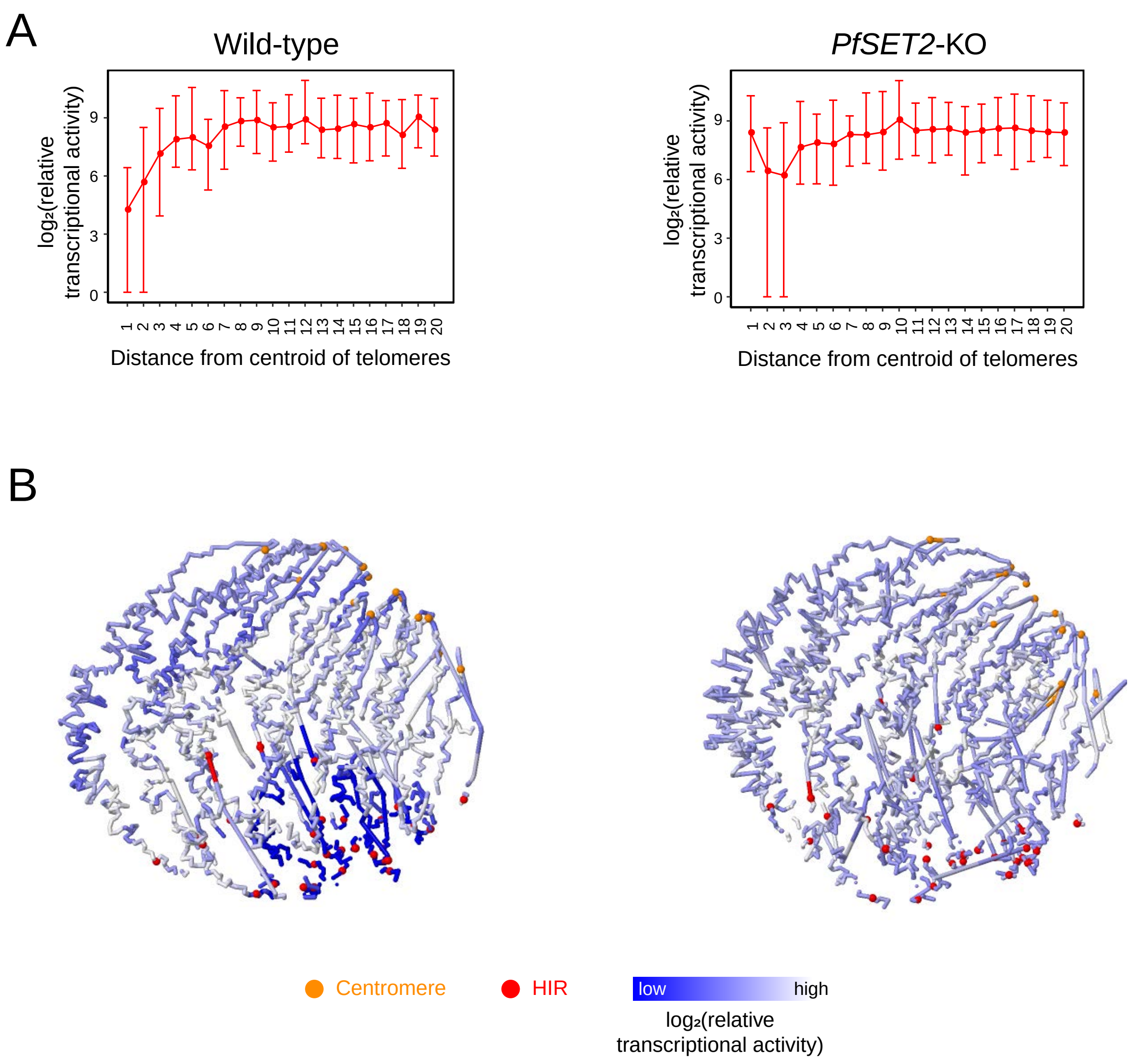

Figure S8: Correlation between genome organization and transcriptional activity. (**A**) Transcriptional activity in regions with increasing distance from the centroid of the telomeres in wild-type and *PfSET2*-KO parasites. The average transcriptional activity of genes was calculated and plotted for each bin. (**B**) Each bin's average transcriptional activity values were colored onto the 3D models. The color scale ranges from blue (low transcriptional activity) to white (high transcriptional activity). Centromeres are indicated with orange spheres and the middle of each HIRs with red spheres.

Figure S9

A

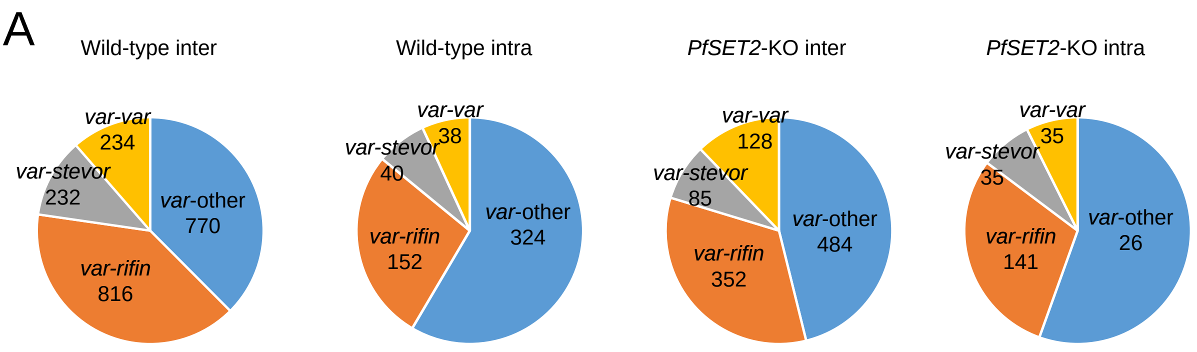

B

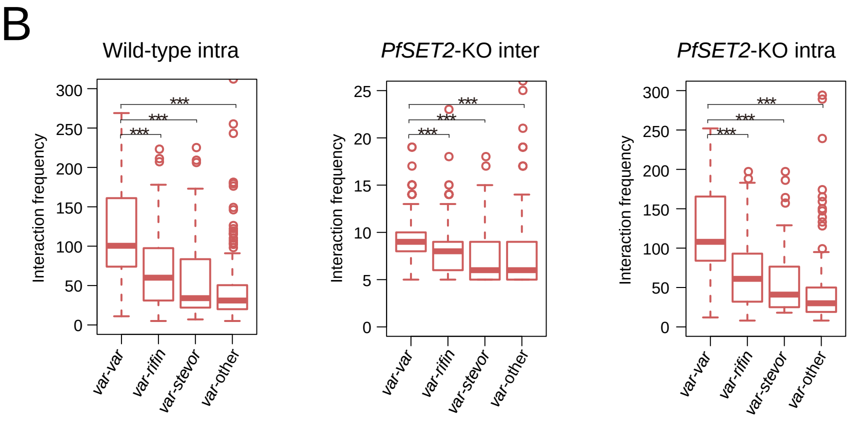

Figure S9. *Var* genes promoter associated interactions. (**A**) Numbers of *var* gene promoter regions associated interactions for intra- and inter- chromosomals in wild-type and *PfSET2*-KO samples. (**B**) Box plots of contacts frequency for promoter associated interactions divided into *var*-to-*var*, *var*-to-*rifin*, *var*-to-*stevor*, *var*-to-other in Wild-type sample.
